# The influence of pulse duration and exposure time of Er,Cr:YSGG laser on lithium disilicate laminate debonding, an *in vitro* study

**DOI:** 10.1016/j.heliyon.2023.e14600

**Published:** 2023-03-15

**Authors:** Sura Sardar Al-Karadaghi, Hussein Jawad, Tamara Al-Karadaghi

**Affiliations:** a Department of Biomedical Applications, Institute of Laser for Postgraduate Studies, University of Baghdad, Baghdad, Iraq; b Orofacial Pain, School of Dentistry, University of California Los Angeles UCLA. 10833 Le Conte Ave, CHS 10-157, Los Angeles, CA 90095, USA

**Keywords:** Er,Cr:YSGG laser, Lithium disilicate veneers, Intrapulpal temperature, Shear bond strength

## Abstract

**Problem statement:**

Grinding restorations, such as veneers, with rotary instruments, is the conventional removal approach. It may be accompanied by micro-fractures that affect the adjacent healthy dental structures. Differentiation of the veneer from the dental structure, as well as the resin cement, is not a highly selective procedure when rotary instruments are used. Moreover, the rotary instruments may lead to scratches and overheating of the enamel. Patient discomfort is another disadvantage, due to the noise of the drill.

**Purpose:**

The purpose of this *in vitro* study was to examine the effectiveness of a 2790 nm Er,Cr:YSGG laser to debond lithium disilicate laminate, utilizing two distinct pulse durations and various exposure times. The shear bond strength, intrapulpal temperature, and adhesive remaining index were evaluated.

**Methods and materials:**

This study included three groups of 75 extracted permanent mandibular incisors: G1, G2 (laser-treated groups were classified according to the pulse duration) and C (control group). Twenty five samples were regarded for each group. Both test groups were irradiated with a 3 W output power of Er,Cr:YSGG laser, for a variety of time intervals (20 s, 30 s, 40 s, 50 s, and 60 s). Five samples were tested for each time interval. During irradiation, the temperature in the pulp chamber was monitored using a K-type thermocouple connected to a digital multilogger thermometer that was introduced into the prepared sample pulp chamber. Subsequently, the shear bond strength was measured for G1 and G2, in addition to the control group (no irradiation). The adhesive remaining index was examined microscopically. The area was measured and analysed, and then, transformed into scores for comparisons. Finally, One untreated sample and two other samples of the highest power value from laser-treated groups were examined for their surface morphology by scanning electron microscope (SEM).

**Results:**

The debonding protocols were safe relative to the intrapulpal protocol. The temperature rise after an exposure time of 50 s and 60 s was significantly different from an exposure time of 20 s, 30 s, and 40 s, in both groups (p < 0.05). Both G1 and G2 significantly outperformed the control group in shear bond strength. There was no significant difference between G1 and G2 at any of the tested exposure times (p > 0.05). Nevertheless, the 60 s exposure time showed the lowest shear bond strength.

**Conclusion:**

Regarding intralpulpal temperature, both modes can be safely used to remove laminate veneers. In sum, an exposure time of 50 s and a pulse duration of 60 μs demonstrated superior results for SBS, adhesive remaining index, and temperature values.

**Clinical implication:**

Lithium disilicate laminate veneers may be removed quickly, safely, and comfortably. Laser-assisted debonding of porcelain laminate veneer is recommended and does not cause any damage to the veneer or enamel surface.

## 1 Introduction

Veneers are popular as a restorative dentistry solution, owing to the enhanced qualities of lithium disilicate. It has slightly better biocompatibility than other types of porcelain and is stronger and better at bonding. The toughest and strongest glass ceramics currently in the market, lithium disilicate glass ceramics (LD), have outstanding shade matching and translucency possibilities despite having moderate flexural strength (360 MPa–440 MPa) [1], and fracture toughness (2.50 MPa–3 MPa) [[2], [3], [4]].

The superior aesthetic and mechanical features of ceramic laminates made of LD and ceramic veneers render them trustworthy as treatment alternatives [5]. Primarily indicated in the anterior regions, these fixed prostheses include onlays, inlays, veneers, full crowns, and other restoration types [6]. Depending on the chosen treatment option, veneer thickness can range from 1.0 mm to 1.5 mm for crowns and 0.3 mm and 1.3 mm for veneers [7].

Secondary caries, colour changes, marginal fractures, and sensitivity are considered major undesirable outcomes after cementation. These adverse outcomes may make it necessary for the restoration to be removed [8].

Grinding these restorations with rotary instruments to remove them is a conventional procedure. The process may be accompanied by microfractures that may affect adjacent dental structures. Differentiation of the veneer from the resin cement and dental structures with rotary instruments is not a highly selective procedure. Moreover, it may result in scratches and overheating of the enamel [9]. Patient discomfort is another disadvantage because most patients are unhappy with the noise of the drill.

Different types of lasers have been used to remove metal-free restorations, including diodes [10,11], CO2 [[12], [13], [14]], Nd:YAG [15], Er:YAG [16], and Er,Cr:YSGG lasers. Erbium lasers have been used effectively for debonding veneers [17,18]. The thickness of the veneer is a crucial factor in selecting the laser power setting for debonding. Veneer thickness is inversely proportional to both the laser power setting and resistance to debonding [19]. Importantly, bonding failure with LD is mainly associated with cohesive failure in the cementing agent, rather than thickness [19].

The main mechanism of action of erbium lasers in veneer debonding is the photoablation or laser softening effect of the resinous cement material. In contrast to Er:YAG, Er, Cr:YSGG has a stronger affinity for hydroxyl groups and hydroxyapatite [20]. When the water content is the primary inhibitor in a specific hard or soft tissue, Er,Cr:YSGG laser has a 3 times lower rate of absorption compared to Er:YAG laser [21,22,55,56].

The chemical composition, ceramic type, and restoration thickness, as well as the type of resin cement, shade, ceramic shade, opacity, and laser parameters such as pulse duration, frequency, power, and time of irradiation, can all impact the clinical outcome of laser-assisted removal of ceramic veneers [[23], [26], [36], [46]].

In a previous study, Alikhasi et al. demonstrated the efficiency of Er,Cr:YSGG for the successful debonding of feldspathic and LD glass-ceramic veneers luted with resin cement on bovine teeth [48].

Until now, there has been a gap in the literature on the impact of various settings of Er,Cr:YSGG lasers including [27] power, pulse duration, and exposure time on veneer debonding relative to tooth structural integrity or breaching the safe threshold and intra-pulpal temperature, as stated by Cohen and Zach [53].

Thus, this study aimed to compare the debonding strength, failure mode (by microscopic examination of debonded enamel surfaces and veneers) and dental pulp temperature were assessed using Er,Cr:YSGG lasers at different pulse durations and exposure times utilizing a standardised size and thickness of LD veneers.

The first null hypothesis was that neither the Er,Cr:YSGG laser nor pulse duration irradiation would affect the shear bond strength of LD ceramic discs bonded to the teeth while the second null hypothesis was assumed that the conventional method offered a safer procedure to debond veneers with pulp and tooth surface damage prevention compared to laser-assisted veneer removal.

## 2 Material and methods

### 2.1 Sample selection

The study sample included 75 extracted bovine permanent mandibular incisors of comparable size and age (2–3 years). All teeth were non-carious, with no crown fractures or enamel defects [34]. The sample size was determined based on a pilot study and previous studies [48]. The teeth were divided into three major groups randomly. The samples were kept in thymol solution (0.1%) for no longer than 2 weeks.

### 2.2 Sample preparation

For each tooth, the organic residues were removed by water steam, while the coronal surface was cleaned with a prophylaxis brush and pumice. A veneers preparation burs kit (Luserdent FG Preparation Set Plus) was used with an electrical handpiece (Coxo) at a standardised speed (200 000 rpm) to prepare the labial aspect of the tooth without exposing the dentine. The same operator prepared all the teeth (five preparations a day), with a self-limiting depth-cutting bur (e.g. a dental FG diamond veneer preparation bur) and a polyvinyl siloxane (silicone index) was used for standardisation to ensure that the tooth reduction and depth of preparation were controlled. A depth preparation bur was used to create 0.7 mm orientation grooves. All the teeth were prepared under a dental microscope (Zumax OMS1951), without any sharp line angles, using a highspeed rotary handpiece, under water coolant.

Gates Glidden #5 and #6 (Dentsply) burs were introduced through the apical foramen to enlarge the root canal in a retrograde manner. All residual pulp remnants were flushed using 2 mL of normal saline.

A 2 mm × 3 mm × 2 mm mould filled with cold-cured acrylic resin (Veracril) was used to prepare each bovine tooth sample. A colour-coding system was adapted for ease of differentiation between the groups. As a separating medium, Vaseline was applied on the moulds before pouring cold-cure acrylic resin, and then, cold-cure acrylic resin was poured into the moulds. Each prepared tooth sample was secured in a mould resin. Subsequently, the sample surfaces were polished and rinsed to remove debris. The prepared moulds were stored in saline to preserve the samples from any effect on their graphene until further use.

### 2.3 Cementation of lithium disilicate laminates

The ceramic veneer squares used in this study were Emax Press HT (LD glass-ceramic high translucency (HT); Ivoclar, Vivadent), in shade A2, with a thickness of 0.7 ± 0.05 mm.

To obtain a uniform thickness, laminate dimensions of 8 mm × 8 mm were used for all groups and prepared in the dental laboratory according to the manufacturer’s instruction, using a digital 3D dental laboratory scanner, then a CAD/CAM design was made. Subsequently, a digital caliper was used to check the thickness and dimensions of each specimen.

Laminate veneers were attached to the labial surfaces of the incisors using light-cured resin cement (Ivoclar Vivadent Variolink Aesthetic LC). All veneers were applied using OptraStick applicators (Ivoclar Vivadent) and etched for 20 s, with a 5% IPS Ceramic Hydrofluoric acid. They were then washed, air-dried, salinised with Monobond N (Ivoclar Vivadent), and allowed to react for 60 s before being cleared of any access with a powerful blast of air. The prepared enamel surface was then scrubbed clean using a polishing brush and fluoride-free paste (Ivoclar, Proxyt, Vivadent), washed with oil-free air, and dried.

A 37% phosphoric acid gel (Ivoclar Vivadent) was applied to the enamel surface for 20 s–30 s. Then, it was rinsed thoroughly with a vigorous stream of water for at least 5 s, and dried with compressed air until a chalky white appearance was achieved. Subsequently, a universal adhesive was applied to the prepared enamel surface and scrubbed for 20 s. They were then air-dispersed and light-cured for 20 s.

As a final step, the laminate was cemented on the tooth structure using an OptraStick applicator (Ivoclar Vivadent), and light-cured in two steps according to the manufacturer’s instructions. After 2 s of exposure, the excess cement material was removed, followed by a final 20 s of light-curing (blue phase, light output of 1200 mW/cm; Ivoclar Vivadent).

To simulate the intraoral condition, all samples were preserved in an incubator filled with distilled water at 37 °C for a period of 48 h [28]. Then, a K-type thermocouple (Amprobe TMD56), with a basic 0.05% accuracy, was inserted into the pulp chamber through the apical foramen.

### 2.4 Sample grouping

Samples were randomly divided into three main groups (n = 25 each), laser-treated groups were classified according to the pulse duration. Each group was subdivided into five subgroups according to the irradiation time (n = 5), as illustrated in Table 1.

**Table 1:** Samples grouping and laser irradiation protocols.

Group	No. of samples	Pulse duration (μs)	Exposure Time (s)	Power (W)	Energy Density (J/cm^2^)
**G1**	**25**	**60**	**20**	**3**	**53**
			**30**		
			**40**		
			**50**		
			**60**		
**G2**	**25**	**700**	**20**	**3**	**53**
			**30**		
			**40**		
			**50**		
			**60**		
**C**	**25**				

Twenty Five samples were assigned as control group (n = 25). No laser irradiation was performed. The control samples provided the baseline for SBS measurement.

### 2.5 The laser system and irradiation protocol

Disk veneer samples were irradiated with an Er,Cr:YSGG laser (2790 nm Waterlase iPlus; Biolase) using a scanning method [[38], [39], [40]]. The temperature was recorded, and the shear bond strength needed to debond the LD veneers was tested immediately after the completion of laser irradiation.

In all groups, a gold handpiece with a 600 μm quartz MZ6 tip (Biolased Technology, Biolase) was utilised in a non-contact mode, at a distance of 2 mm perpendicular to the veneer surface, using distilled and deionised water at a 60%–40% air to water ratio for irrigation. A pilot study was conducted to identify the optimal parameters, and the ensuing irradiating procedure was chosen based on the pilot results and previous studies, as shown in Table 1 [48,49].

In the first group (G1), samples were irradiated using 3 W of power and 60 μs pulse duration (referred to as the H mode in the manufacturing settings), at different exposure times (20 s, 30 s, 40 s, 50 s, and 60 s), and energy density (53 J/cm^2^). In the second group (G2), samples were irradiated using the same power setting (3 W), energy density (53 J/cm^2^), and 700 μs pulse duration (referred to as the S mode in the manufacturing settings), at different exposure times (20 s, 30 s, 40 s, 50 s, and 60 s). Laser irradiation was performed using a scanning method with horizontal movements parallel to the surface, as indicated by Oztoprak et al. [29].

## 3 Measurements

### 3.1 Shear bond strength test

A shear bond strength (SBS) test was conducted on the control samples (the control group), and the test groups (G1 and G2) n = 25 each, to determine changes in bond strength after laser radiation of the test groups relative to the control group.

Each sample was mounted on a universal testing device (Beijing Jinshnengxin), for the shear test. At the tooth veneer interface of the tested tooth, a 0.5 mm chisel blade was placed. At a crosshead speed of 0.5 mm/min, a shear force was delivered in the form of a static load to remove the veneer. Prior to sample failure, all data were logged in MPa.

The maximal failure load in N (Newton) was recorded and converted to MPa by dividing the failure load by the bonding area (64 mm), as illustrated in equation (1).

Where: Area = exacted diameter of the bonded surface

(1) $$Shear\;stress\;\left(MPa\right)=\frac{Load\;\left(N\right)}{Area\;\left({mm}^{2}\right)}$$

All the results were electronically recorded using a computer entry system (Lenovo, AMD Radeon Graphics).

### 3.2 Dental pulp temperature

For both the test groups (G1 and G2), the intra-pulpal temperature was continuously recorded using a K-type thermocouple (Amprobe TMD-56).

A K-type thermocouple was inserted into the pulp chamber through the apical foramen. To ensure that the tip of the thermocouple touched the highest location of the pulp chamber, a verification radiograph was taken. After the thermocouple was placed inside the pulp, the root apex was sealed with composite resin (Tetric N-Ceram, Ivoclar Vivadent) [30]. The root portion of the sample was submerged in a water bath to simulate the *in vivo* scenario and increase the transmission of the generated heat during the laser irradiation. The tooth with the attached thermocouple was secured in place using a holder. The starting temperature was 36.6 ± 1 °C. Data were logged to a PC using an Amprobe TMD-56 digital multilogger thermometer and a USB bus controller coupled to software (Amprobe multiline V3.0).

### 3.3 Microscopic analysis

The bond failure mode was categorised into three types according to the modified criteria and the adhesive remnant index (ARI) score, as illustrated in Table 2.

**Table 2:** Scores Adhesive Remnant Index as described by Artun [54].

	
0	No adhesive remained on enamel
1	Less than 50% of the adhesive remained on enamel
2	More than 50% of the adhesive remained on enamel
3	All adhesive remained on enamel

Digital photographs of each specimen and each tooth surface after failure were taken under a universal testing machine using a stereomicroscope under ×4 magnification (Olympus B×51).

All photographic results were digitalised and analysed using software, and all data were collected, tabulated, and statistically analysed.

### 3.4 Scanning electron microscope (SEM)

One untreated sample and two other samples of the highest power value from laser-treated groups were examined for their surface morphology by coating their bonding surfaces with gold-palladium (Rotary Pumped Sputter Coater, CYSI) and then observed under SEM (Thermo Fisher Scientific Axia).

## 4 Results

**Fig. 1:**
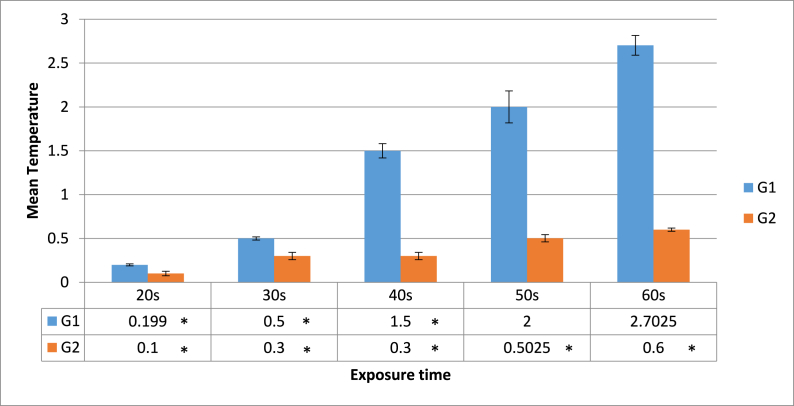
Mean increment of the pulpal temperature (±SD) during laser irradiation of laminate samples. Experimental groups: short pulse duration laser group (G1) and long pulse duration (G2), each tested for 20s, 30 s, 40s, 50s, 60s exposure time. *Significantly different when compared with G1/50s and G1/60s by a post hoc Tukey test with (p < 0.05).

Fig. 1 displays the mean and standard deviation of the temperature increase throughout each irradiation protocol. In general, specimens in the G1 group had a greater temperature increase than those in the G2 group during laser irradiation, according to the mean increment in pulpal temperature. By increasing the laser exposure time in both groups from 20 s to 60 s, a direct correlation between the exposure time and temperature was found. The largest temperature increase during laser-assisted debonding was observed with G1/50 s and G1/60 s, in which the temperature increased by 2 °C and 2.7 °C, respectively. These temperatures were below the cut-off for pulpal damage. According to Fig. 1, irreversible damage to pulpal vitality was unlikely with any of the laser irradiation protocols.

**Fig. 2:**
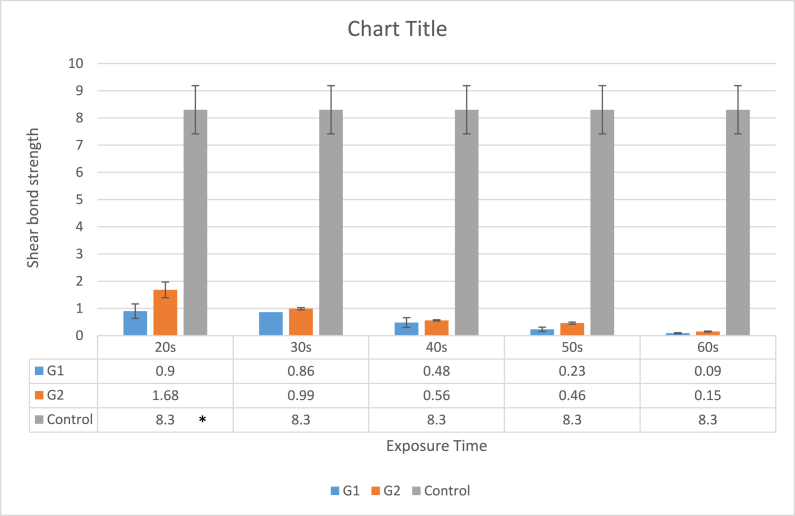
Mean SBS (±SD) during laser irradiation of laminate samples. Experimental groups: control group, short pulse duration laser group (G1) H mode and long pulse duration (G2) S mode, each tested for 20s, 30 s, 40s, 50s, 60s exposure time.*Significantly different when compared with G1 and G2 by a post hoc Tukey test with (p < 0.05).

**Fig. 3:**
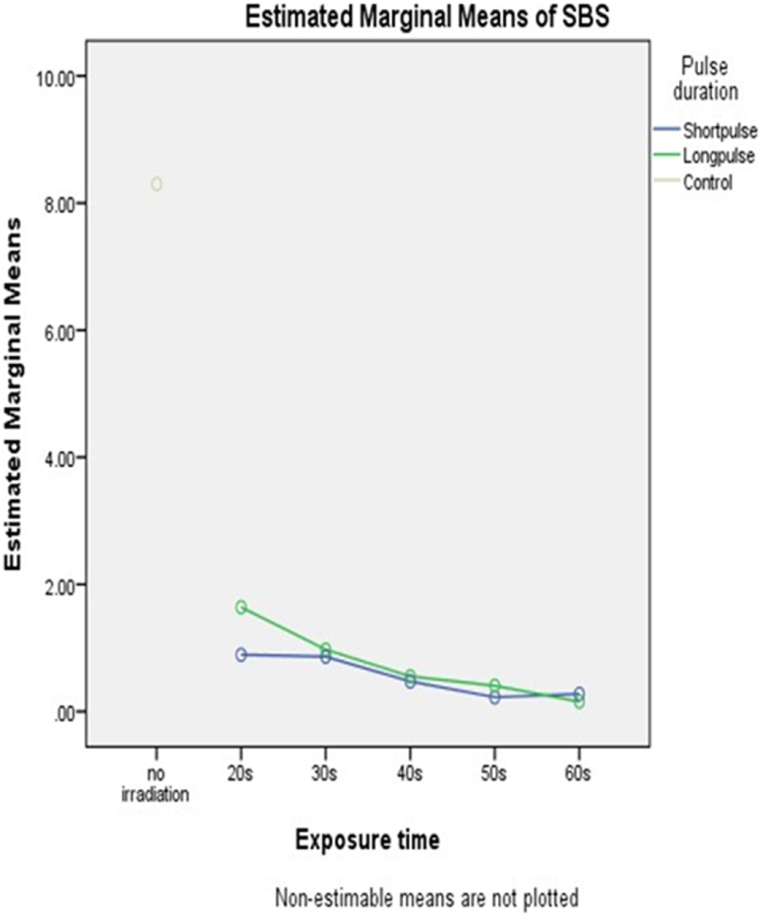
Exposure time versus SBS reduction during the different exposure times.

All data were normally distributed according to the Shapiro-Wilk normality test. ANOVA and post-hoc Tukey test were employed. With a p-value of 0.00002, ANOVA showed a significant difference between the groups. The G1 time considerably outperformed the other test groups (p < 0.016), in terms of temperature increase after 50 s and 60 s of exposure. The temperature increase between the G2 subgroups and G1/20 s and G1/30 s did not differ significantly (p > 0.05). Regardless of the laser pulse duration and exposure time, there was a substantial difference in the SBS between the control group and both the test groups (p < 0.00026). (Fig. 2, Fig. 3). There were no significant differences between G1 and G2 in terms of exposure time (for all exposure time settings) (p > 0.05). However, a 60 s exposure time was associated with lower SBS.

**Fig. 4:**
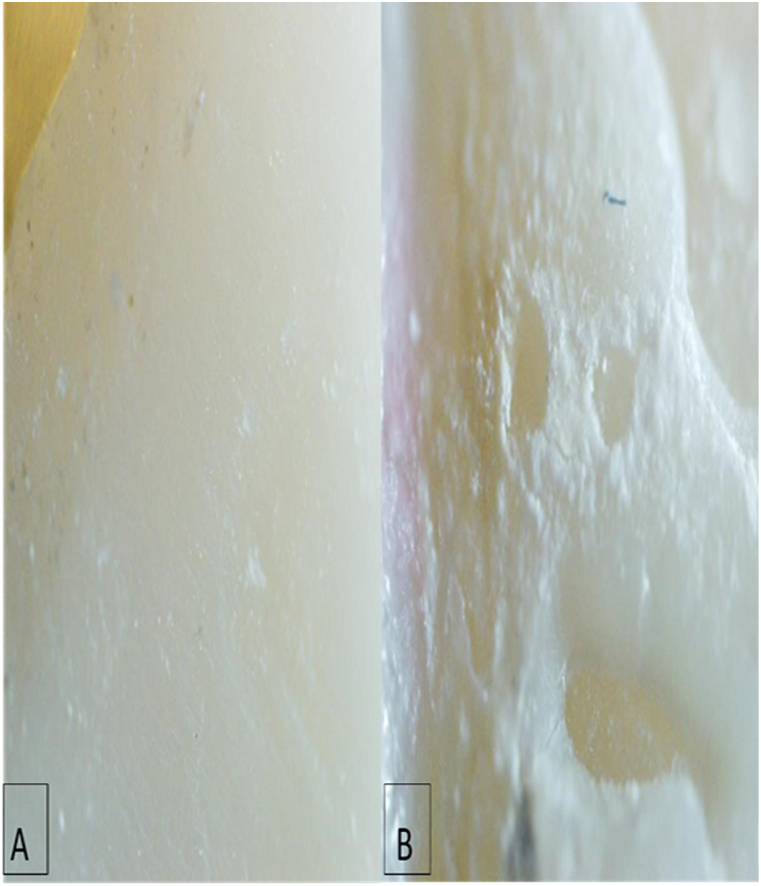
Stereomicroscope pictures of A-veneer surface with spots of cement remaining. B- enamel surface of tooth structure with the most adhesive remnant cement.

Most of the specimens demonstrated an ARI score of 2, with the exception of four samples which had a score of 3, as shown in Fig. 4 (A, B).

**Fig. 5A:**
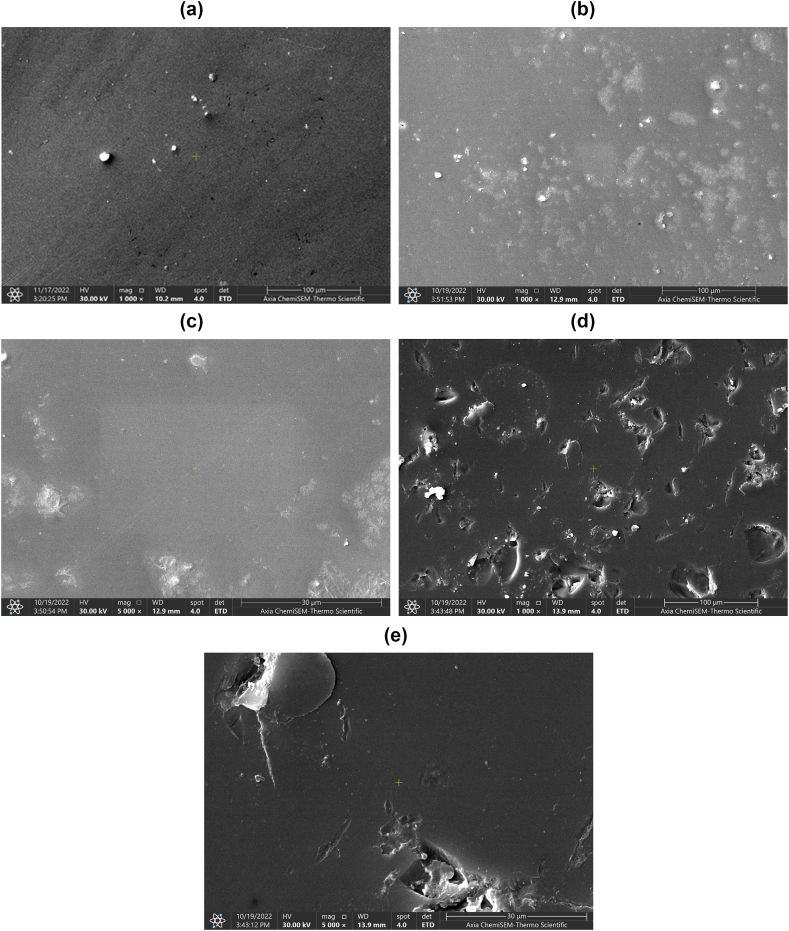
SEM images of LD veneers specimens with gold coating view of untreated (control) specimen surface with 1000× magnification. SEM images of LD veneers specimens with gold coating view of laser irradiated group (Gold handpiece with 60 μs pulse duration) specimen surface with 1000× magnification. SEM images of LD veneers specimens with gold coating view of laser irradiated group (Gold hand piece of 60 μs pulse duration) specimen surface with 5000× magnification. SEM images of LD veneers specimens with gold coating view of laser irradiated group (Gold hand piece of 700 μs pulse duration) specimen surface with 1000× magnification. SEM images of LD veneers specimens with gold coating view of laser irradiated group (Gold handpiece of 700 μs pulse duration) specimen surface with 5000× magnification.

The SEM pictures of the LD veneer disc surface are illustrated in Fig. 5(A–E). The texture of the surface in Figure (5A) shows the non-treated sample surface morphology and (B-E) shows those specimens that were treated with the laser irradiation by gold handpiece which exhibited a morphological irregularity just like holes spots made by the irradiation pulses pattern that is unique for the applied laser of the gold handpiece but without any micro or macrocracks, the visualized micro holes had no cracks, fissures nor charring. The created micro irregularities differ for the applied laser parameters, for the two examined laser-treated specimens, which could be detected easily on the veneer surface with 1000 and 5000 magnifications.

## 5 Discussion

In this study, the debonding effect of irradiation using an Er,Cr:YSSG laser on LD veneers was observed. The study also measured the resistance to debonding and the amount of adhesive cement residue on the veneer and tooth surface. Other researchers have also previously investigated the effectiveness of lasers for the debonding of various restorative materials [5,16,28,31,32].

One of our concerns was the amount of laser light that was delivered to the pulp when the temperature increased during debonding and the exposure time also increased. In contrast, the fabrication material and thickness, were comparable to the ones employed by Sari et al. Moreover, Sari et al. evaluated the laser light transmission through various ceramic materials, including sintered zirconium oxide ceramics, feldspathic ceramics, LD ceramics, leucite-reinforced glass ceramics, and monolithic zirconium oxide ceramics. There were two distinct thicknesses in each group: 0.5 mm and 1 mm. The results showed considerable differences between the various materials. For example, 88% of LD ceramics with 0.5 mm thickness achieved the maximum laser transmission [32]. Following a primary pilot study and a manufacturing set-up, the laser irradiation methodology was chosen as the power setting. This upper limit used in this study is consistent with that reported by Gurney et al., who employed an Er,Cr:YSGG laser to determine the ideal power and exposure time for the removal of LD veneers. They discovered that veneers can be removed without causing pulpal damage when utilizing 3.5 W and 4 W of output powers, applied for 60 s [16]. When employing an Er,Cr:YSGG laser in clinical practice, this approach is recommended for debonding veneers of various thicknesses [18,33,34].

Considering limiting experimental errors, freshly extracted bovine teeth were selected for this study. Previous studies support the use of bovine teeth due to histological and physical similarities between bovine teeth and human teeth, and there is sufficient literature to justify their usage [[14], [15], [16]].

Tocchio et al. [35] described various debonding methods that allow the adhesive resin cement to degrade by laser energy as thermal ablation, thermal softening, or photoablation. The scanning approach and the laser debonding protocol utilised in the current investigation did not result in any explosive "blow-offs" that would indicate thermal ablation and photoablation. However, given that methacrylate is a target chromophore, it is logical to anticipate physical disruptions as opposed to thermal softening of the luting composite. Considering this reason, it is logical that by lengthening the exposure time, the debonding process is hastened.

According to Oztoprak et al., laser-assisted laminate veneer removal was more likely to be related to thermal ablation and photoablation, than explosive “blow-offs”, because laminate veneer removal through the scanning approach did not result in it. In that study, the authors proposed that physical disruption in the resin cement may be another underlying degradation mechanism, and suggested that this process did not occur solely as a result of thermal softening [9]. Instead of thermal softening leading to debonding, Morford et al. defined this mechanism as laser ablation [28].

Based on these investigations, we propose that the underlying mechanisms of porcelain laminate veneer removal include thermal softening, in addition to cement ablation, physical disruption, and cement ablation. The debonding mechanism of failure is an indicator of enamel damage. The risk of enamel damage is increased by failure at the enamel-cement interface. In our research, using microscopic images, the failure occurred at the veneer cement interface, and almost all resin cement remained on the tooth surface, which highlights the conservative side of veneer debonding by laser. This result is consistent with the findings of Morford et al. who reported that most failures occurred at the veneer-cement interface. However, the veneer surfaces have not been investigated. The results of our study concurred with Morford et al. In both studies, laser-assisted removal of the porcelain laminate veneers could be accomplished without damaging the enamel [2]. In line with more recent studies [5,32], we also showed that laser irradiation did not have any detrimental effects on ceramic surfaces. In particular, no cracks or fractures were observed in the microscopic images.

An increase in the pulpal temperature throughout laser irradiation is one of the main issues associated with laser-aided debonding. Zach and Cohen [53] first indicated in 1965 that a pulpal temperature increase of over 5.5 °C is detrimental to pulpal health. Our findings suggest that depending on the irradiation time, laser power, and pulse duration, the Er,Cr:YSGG laser parameters utilised in this study can lead to a range of temperatures. The mean temperature increase was less than 3 °C in all the specimens, with speciments in the G2 group experiencing the largest increase (2.8 °C). This demonstrates that LD can be reused after being removed using Er,Cr:YSGG, without losing any structural properties. These results are in line with those of earlier studies on implant crowns [26,37,38,50,51]. The scanning technique [29,39,40] was utilised, which could be one of the most significant factors for the low-temperature increase. To reduce the likelihood of thermal damage, the laser type, settings, cooling air, and water sprays can all play a role. The ratio was 40%–60% water.

To our knowledge, this is the first study to report the optimal effects of applying Er,Cr:YSGG lasers at different pulse durations to the debonding process of porcelain laminate veneers. Based on pulse duration, some studies stated that higher temperature and penetration values were observed in the 60 μs group, which were accompanied by lower SBS values. This could be because decreasing the pulse duration is usually accompanied by a higher peak power. By increasing the pulse duration heat dissipation following laser irradiation may also be increased, which may adversely affect the strength of the ceramic structures. Thus, both pulse duration and power setting contribute a conceding effect on temperature elevation, and subsequently, on SBS [,[24], [25], [41], [42]]. The results of this study concurred. In this study, the maximum increase in temperature with the short pulse duration G1 was 2.8 °C, while the long pulse duration G2 was 0.6 °C.

Based on the results, the veneers of the laser groups required considerably less shearing force to debond compared with that of the control group. Laser application significantly reduced the required debonding load for the laminate veneers, and there were no significant differences between the two pulse durations used in the debonding process in this study.

This study aimed to minimise the retrieval time at the lowest possible laser settings to prevent potentially dangerous temperature spikes and irreparable pulp and enamel injuries. Various laser settings for debonding repair have been suggested by different researchers. In the pilot study, we tested multiple parameters, chosen from a published protocol, for the retrieval of all-ceramic crowns and veneers, in an effort to determine the optimum efficiency relative safety [43,44].

In terms of laser exposure time, 50 s showed the most satisfactory results in both intrapulpal temperature elevation and SBS. However, at 60 μs (H-mode), a superior profile was observed compared to a longer pulse duration of 700 μs (S mode).

Al-Maajoun et al. [45] also reported a statistically significant difference between the control group and both laser groups in the SBS of E-max ceramic restorations following laser irradiation, which is consistent with the results of the present study. In this study, we found that even at the lowest exposure time of 20 s, there was a jump in the SBS. In a study conducted by Nalbantgil et al. [9], ceramic brackets were debonded from the enamel surface by Er:YAG lasers with a significant decrease in SBS, which is consistent with the findings of this study.

Nalbantgil et al. [9] found that laser irradiation time impacted SBS considerably. As the laser irradiation time increased, the amount of force required for debonding decreased. During laser debonding, the irradiation energy passes through the ceramic to the resin cement, which absorbs the remaining energy without changing the chemical composition of the tooth surface or the ceramic surface [5,18,28,44,47].

In the present study, no colour changes were observed in the enamel following laser irradiation. The results are consistent with the findings of Nalbantgil et al. [9] and Oztoprak et al. [29] Moreover, Oztoprak et al. observed localised carbonisation and black deposits in the residual resin cement, and not in the tooth structure. Furthermore, Reichmann et al. [47] observed no discoloration in the dentine bonding zone.

According to SEM pictures, the difference in the pattern of a variant laser pulse irradiation that was shown during SEM analysis illustrates a preferable incline toward shorter pulse duration, this comes into agreement with Fateima et al. [52] who concluded that the 60 μs laser pulse duration is better than the 700 μs.

This study has a few limitations. Only one type of cement, one veneer thickness (0.7 mm), and one dimension (8 mm × 8 mm) were tested. The results may vary depending on the type of cement used and the cementation process utilised. In the future, *in vivo* experiments that assess different retrieval times, cementation techniques, material thicknesses, and laser handpieces are warranted. Furthermore, clinical difficulties and patient perceptions should be considered.

## 6 Conclusion

The results showed that LD laminate veneers can be removed effectively, quickly, and safely using Er,Cr:YSSG lasers at different pulse durations (H and S mode), as well as a varied irradiation time. The SBS of the porcelain laminate veneers was reduced by lengthening the pulse duration from extremely high values to those at which removal was simple. Irrespective of the irradiation technique, the intrapulpal temperature caused by laser irradiation was within the physiological range, and unlikely to cause any negative effects on the dental pulp or tissues in close proximity.

## Author contribution statement

Sura Sardar Al-Karadaghi: Performed the experiments; Contributed reagents, materials, analysis tools or data; Wrote the paper.

Hussein Jawad: Conceived and designed the experiments; Analysed and interpreted the data; Contributed reagents, materials, analysis tools or data.

Tamara sardar Al-Karadaghi: Analysed and interpreted the data.

## Funding statement

This research did not receive any specific grant from funding agencies in the public, commercial, or not-for-profit sectors.

## Data availability statement

Data will be made available on request.

## Declaration of interest’s statement

The authors declare no conflict of interest.
